# Psychometric Evaluation of the Lithuanian Version of the Revised Compound Psychological Capital Scale (CPC-12R) and Its Associations with Nature Exposure and Physical Activity

**DOI:** 10.3390/bs16081454

**Published:** 2026-08-21

**Authors:** Migle Baceviciene, Vaiva Balciuniene, Rasa Jankauskiene

**Affiliations:** Health Research and Innovation Science Center, Faculty of Health Sciences, Klaipeda University, 92294 Klaipeda, Lithuania; vaiva.balciuniene@ku.lt (V.B.); rasa.jankauskiene@ku.lt (R.J.)

**Keywords:** psychological capital, CPC-12R, scale validation, nature exposure, physical activity in nature, mental health, Lithuania

## Abstract

Psychological capital (PsyCap), comprising optimism, hope, self-efficacy, and resilience, is an important positive psychological resource associated with well-being and adaptive functioning. The present study aimed to evaluate the psychometric properties of the Lithuanian version of the revised Compound Psychological Capital Scale (CPC-12R) and to examine its associations with nature exposure and physical activity. A cross-sectional survey was conducted among 1255 Lithuanian adults aged 18–70 years (mean age 39.51 ± 13.84 years), and 64.4% were women. The total sample was randomly divided into exploratory (*n* = 628) and confirmatory factor analysis (*n* = 627) subsamples. Exploratory factor analysis supported a two-factor structure separating optimism from the remaining CPC-12R dimensions, whereas confirmatory factor analysis supported the theoretically expected second-order model comprising optimism, hope, self-efficacy, and resilience as first-order factors reflected by a higher-order PsyCap construct. The second-order model demonstrated good fit (robust CFI = 0.963, robust TLI = 0.952, robust RMSEA = 0.074, SRMR = 0.042) and strict measurement invariance across gender and place-of residence groups. Internal consistency was satisfactory to good for all subscales (McDonald’s ω = 0.79–0.85) and excellent for the total scale (ω = 0.93). Evidence of convergent validity was demonstrated through positive associations with self-esteem and life satisfaction and negative associations with psychological distress and poorer mental health. Furthermore, PsyCap explained a small but significant proportion of additional variance in mental health difficulties beyond self-esteem, life satisfaction, and psychological distress. PsyCap was positively associated with nature exposure, physical activity in nature, and leisure-time exercise. Hierarchical regression analyses indicated that greater nature exposure and more frequent physical activity in nature were independently positively associated with PsyCap after accounting for sociodemographic characteristics, whereas leisure-time exercise was not. Overall, the findings support the reliability and validity of the Lithuanian CPC-12R, particularly its total score, although the EFA results suggest caution when interpreting the four subscale scores separately. The cross-sectional findings further identify engagement with natural environments as a meaningful behavioural correlate of PsyCap.

## 1. Introduction

Psychological Capital (PsyCap) emerged from the Positive Organizational Behaviour (POB) movement and has become a prominent construct in both organizational research and the broader field of positive psychology (Luthans et al., 2004; Luthans & Youssef-Morgan, 2017). It was developed to capture a set of positive psychological resources that are open to development and contribute to improved performance and functioning. PsyCap is defined as an individual’s positive psychological state of development characterized by four well-known psychological resources: hope, efficacy, resilience, and optimism (Luthans & Youssef-Morgan, 2017). Hope refers to the ability to pursue goals and find alternative ways to achieve them when obstacles arise. Efficacy reflects confidence in one’s ability to successfully perform challenging tasks. Resilience refers to the capacity to recover and adapt when faced with difficulties or setbacks. Optimism involves maintaining positive expectations about success in the present and future. Together, these four resources form a higher-order construct and are commonly referred to by the acronym HERO (Hope, Efficacy, Resilience, and Optimism). Rather than operating independently, the four components are theorized to interact synergistically, creating a broader psychological capacity that enables individuals to cope with challenges, pursue goals effectively, and maintain well-being (Youssef-Morgan & Luthans, 2015). Accordingly, the four dimensions remain conceptually distinct first-order factors, whereas the higher-order PsyCap construct captures the shared variance underlying these interrelated psychological resources.

PsyCap is different from human and social capital in that it focuses on an individual’s internal psychological resources rather than their knowledge or relationships. While human capital reflects what a person knows (skills, education, experience) and social capital reflects who a person knows (networks and connections), PsyCap refers to who a person is and can become, encompassing positive, developable HERO capacities (Luthans & Youssef-Morgan, 2017). Review studies consistently show that PsyCap is strongly and positively related to desirable work outcomes such as better job performance, higher job satisfaction, well-being, stronger organizational commitment, and lower negative outcomes (Avey et al., 2011; Larson & Luthans, 2006; Luthans et al., 2010; Orgambídez et al., 2025; Yuan et al., 2023). However, the relevance of PsyCap extends well beyond occupational settings. A growing body of research has linked higher levels of PsyCap to a range of positive outcomes, including greater mental health and subjective well-being (Bertieaux et al., 2024; Paul et al., 2026; Preston et al., 2023), life satisfaction (Datu & Valdez, 2019; Delhey et al., 2023; R. Zhang et al., 2019), self-esteem (Zhao et al., 2025), academic outcomes (Li et al., 2023) as well as reduced psychological distress, anxiety, and depressive symptomatology (Liu et al., 2026; Song & Song, 2024). These associations suggest that PsyCap functions as a protective psychological resource, buffering individuals against the negative effects of stress and adversity and promoting adaptive functioning across various life domains.

Given its theoretical and practical importance, the accurate and reliable measurement of PsyCap is essential. To operationalize PsyCap, Luthans and colleagues developed the Psychological Capital Questionnaire (PCQ), which assesses the four HERO dimensions as manifestations of a higher-order PsyCap construct (Luthans et al., 2007). Since its development, the PCQ has been translated into multiple languages and validated across diverse cultural and occupational contexts, demonstrating generally good psychometric properties and supporting the cross-cultural relevance of the PsyCap construct (Dawkins et al., 2013; Newman et al., 2014). Although PCQ is the most widely used measure of PsyCap, several limitations have been noted. Some items were originally developed for organizational settings and may require adaptation for use in other contexts, such as education or health (Dudasova et al., 2021; Lorenz et al., 2016). In addition, evidence regarding the psychometric properties of translated versions was not equally robust across all languages and cultures (Dawkins et al., 2013). To overcome these limitations, Lorenz et al. (2016) developed Compound PsyCap Scale (CPC-12) (Lorenz et al., 2016). Initial evidence supporting the convergent, discriminant, and factorial validity of the CPC-12 was reported by its developers. However, during the adaptation of the scale into another language, Dudasova et al. (2021) identified several psychometric concerns. Although the theoretical four-factor structure comprising hope, self-efficacy, resilience, and optimism was supported in the original German validation study, subsequent analyses revealed considerable overlap between resilience and self-efficacy. In the Czech adaptation, the hypothesized hierarchical model with four first-order factors was not supported; instead, a more parsimonious three-factor model in which resilience and self-efficacy were merged into a single factor provided a better representation of the data. Secondary analyses of the two original German samples revealed the same discrepancy. These findings raised concerns about the factorial distinctiveness of resilience and self-efficacy and led to the development of the CPC-12R with newly formulated resilience items (Dudasova et al., 2021).

Furthermore, the CPC-12R has demonstrated sound psychometric properties across several cultural contexts including Slovak, German, English (U.S.), Arabic, Japanese and Urdu translations (Hussain & Habib, 2026; Ikeda et al., 2022; Kačmár et al., 2022; Lorenz et al., 2022; Mansouri & Lorenz, 2025). Also, measurement invariance was established between German and English translations (Lorenz et al., 2022) and between the U.S., the Czech and Slovakia samples (Prochazka et al., 2025). However, to date, a validated Lithuanian-language version of the CPC-12R has not been reported in the literature, representing a gap that limits the study of PsyCap in this population. Lithuania presents a particularly relevant context for the study of PsyCap and its correlates. As a post-Soviet Baltic state that has undergone substantial social, economic, and institutional transformation over recent decades, the Lithuanian population faces a distinctive profile of psychosocial stressors and resilience-related challenges (Dirzyte et al., 2021). Validating a reliable and theoretically grounded measure of PsyCap in this context is therefore both scientifically and practically warranted. Furthermore, although PsyCap is increasingly conceptualized as a domain-general psychological resource and has been examined across diverse settings, including education, health, and sport (Huang et al., 2025; Kim et al., 2017; Li et al., 2023; Luthans & Youssef-Morgan, 2017; Youssef-Morgan & Luthans, 2015), the CPC-12R has primarily been validated in employee samples (Dudasova et al., 2021; Ikeda et al., 2022; Lorenz et al., 2022; Prochazka et al., 2025). Consequently, it remains important to establish whether the scale demonstrates satisfactory psychometric properties in the general population. Validating the CPC-12R in a non-occupational sample would therefore extend existing evidence regarding its applicability beyond workplace settings and support broader research on psychological resources and well-being.

Beyond measurement, understanding the behavioural and environmental correlates, and particularly the antecedents, of PsyCap is of considerable scientific interest (Luthans & Youssef-Morgan, 2017), as such knowledge may inform the development of interventions designed to strengthen positive psychological resources. PsyCap is thought to exert its effects through several interconnected mechanisms. It promotes goal-directed action, persistence, and a sense of personal agency when facing challenges; encourages more constructive and adaptive interpretations of difficulties; generates positive emotions such as hope, confidence, and optimism that broaden thinking and facilitate resource development; and is reinforced through supportive social relationships that foster resilience and effective coping. Together, these mechanisms enhance individuals’ capacity to pursue goals, overcome adversity, and maintain well-being (Luthans & Youssef-Morgan, 2017).

One environmental factor that has received growing attention in this context is exposure to natural environments (Nejade et al., 2022; Twohig-Bennett & Jones, 2018). A substantial body of research has linked nature exposure to a range of psychological benefits, including reduced stress, improved mood, enhanced cognitive functioning, greater subjective well-being (Banwell et al., 2024; Christiana et al., 2021; Eime et al., 2013; Jimenez et al., 2021; Mensah et al., 2016; Swami et al., 2024; White et al., 2021; F. Zhang & Qian, 2024). Theoretical perspectives such as Attention Restoration Theory and Stress Recovery Theory suggest that natural environments facilitate psychological restoration by alleviating cognitive fatigue and reducing physiological stress responses (Kaplan, 1995; Ulrich et al., 1991). Given that PsyCap is conceptualized as a malleable, state-like psychological resource rather than a fixed personality trait (Luthans & Youssef-Morgan, 2017), it is reasonable to expect that repeated engagement with restorative natural environments may support the development and maintenance of PsyCap, particularly its resilience and optimism components. Despite these theoretical links, empirical evidence directly examining the association between nature exposure and PsyCap remains limited.

Physical activity represents another potentially important correlate of PsyCap. Regular engagement in physical activity has been associated with improved mental health through mediators such as self-esteem, self-efficacy, physical self-worth, body image satisfaction, resilience, social support, social connection, physical health, pain, and fatigue, suggesting pathways through which exercise may strengthen key PsyCap dimensions. Of particular interest is physical activity conducted in natural settings, such as walking or exercising in forests, parks, or other green and blue spaces. Such nature-based physical activity may confer additional psychological benefits over and above those associated with indoor or urban exercise, by simultaneously providing the restorative effects of natural environments and the well-being benefits of physical activity (Peddie et al., 2024). Yet, the extent to which physical activity in nature, as distinct from general leisure-time exercise, is associated with PsyCap has not been empirically examined.

The first objective was to evaluate the psychometric properties of a Lithuanian adaptation of the CPC-12R, including its factor structure, internal consistency, measurement invariance across gender, education and place-of-residence groups, and convergent and incremental validity. The second objective was to examine the associations between PsyCap and nature exposure, physical activity in nature, and general leisure-time exercise in a large community sample of Lithuanian adults, and to assess whether these nature- and activity-related variables were independently associated with PsyCap after accounting for sociodemographic characteristics. We hypothesized that Lithuanian translation of the CPC-12R would replicate the original structure and demonstrate acceptable psychometric properties. Also, we hypothesized that nature exposure and physical activity in nature would be positively correlated with PsyCap controlling for demographic variables.

## 2. Materials and Methods

### 2.1. Participants

The final sample, after applying the inclusion criteria described in the Procedures section, comprised 1255 Lithuanian adults aged 18–70 years (M = 39.5, SD = 13.8). The sample included women and men and varied in educational attainment, marital status, place of residence, and perceived financial security. Detailed sociodemographic characteristics of the study sample are reported in the Results section and presented in Table 1.

### 2.2. Procedures

Data for this cross-sectional survey were collected from April 2025 to February 2026 using an online SurveyMonkey questionnaire. A non-probabilistic volunteer sampling method was used to recruit a broad sample of Lithuanian adults across gender and age groups. The survey link was disseminated through sponsored Facebook advertisements targeting the main municipalities of Lithuania and was also sent to major business companies and public-sector organizations in Lithuania. Participation was voluntary, and no remuneration was provided.

**Figure 1:**
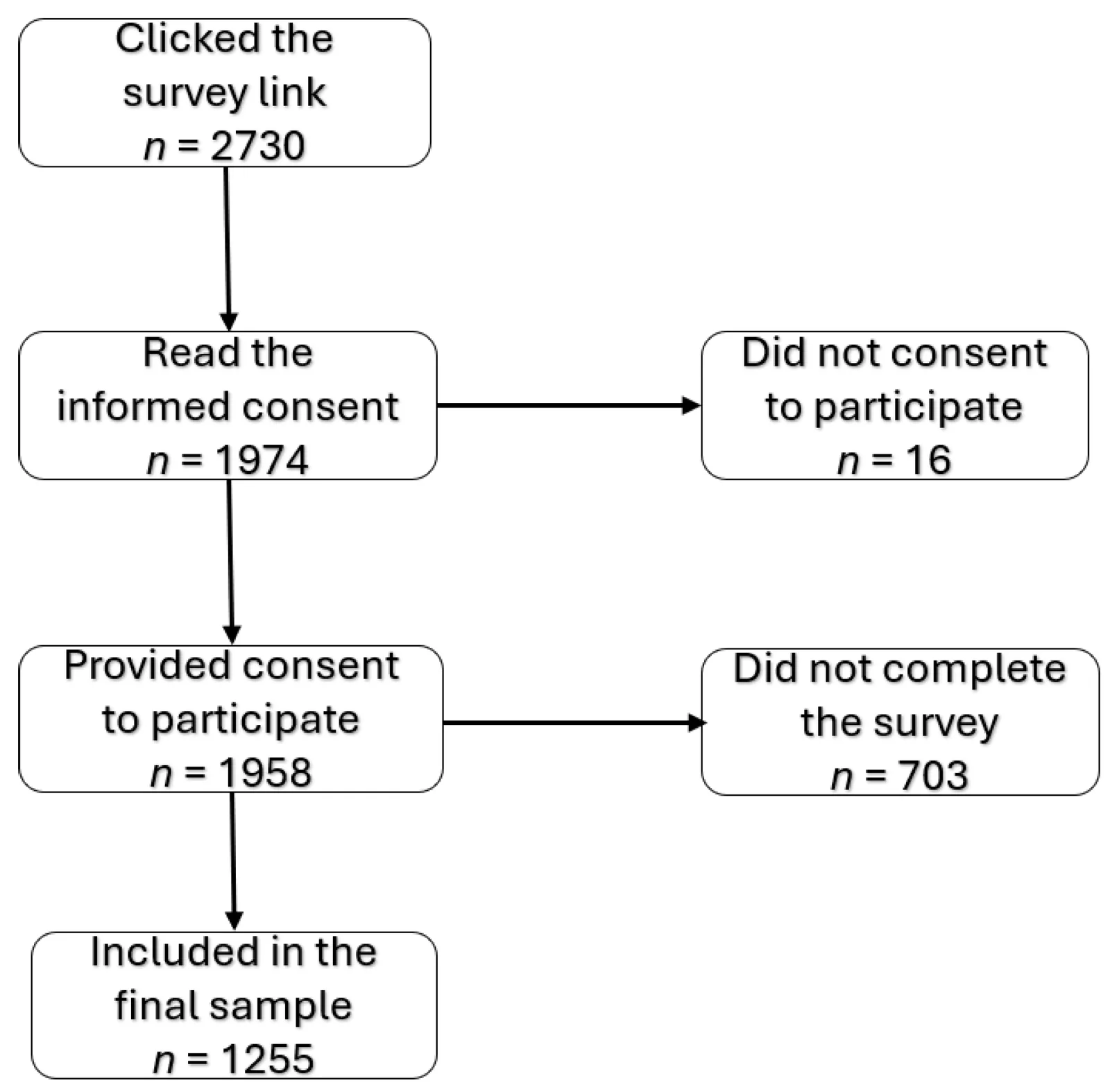
Flowchart of participant recruitment and inclusion in the final analytical sample.

A total of 2730 individuals clicked the survey link, of whom 1974 proceeded to the informed consent information. Sixteen individuals did not provide consent, leaving 1958 participants who consented to participate. Of these, 703 did not complete the survey, typically discontinuing after the sociodemographic section or additionally completing only one or two scales. The remaining 1255 participants completed the survey and were included in the final analytical sample (Figure 1).

Eligible participants were adults aged 18 years or older who were able to complete the questionnaire in Lithuanian. Before completing the survey, participants were informed about the study aims, the research team, and the estimated questionnaire completion time of approximately 20–25 min. In addition, information regarding survey anonymity, confidentiality, voluntary participation, and the use of aggregated data for scientific purposes only was provided. Participants could either provide electronic consent to participate or decline participation. After providing electronic consent, respondents were given access to the study measures, whereas those who declined were thanked, and the survey was terminated. Participants could also stop completing the online survey at any time by closing the browser. The online survey was programmed to require responses to all study questions. Only submitted questionnaires that met the inclusion criteria and contained complete responses were included in the final dataset.

The Lithuanian adaptation of the revised Compound Psychological Capital Scale (CPC-12R) described by its developers as a free-to-use measure of PsyCap (Dudasova et al., 2021). The Lithuanian adaptation of the CPC-12R was prepared following the instrument translation procedure described by Prieto (Prieto, 1992). First, the original English items were independently translated into Lithuanian by two bilingual translators having expertise in psychology/behavioural sciences. The two forward translations were compared, and any discrepancies were discussed and resolved by consensus to produce a preliminary Lithuanian version. This version was subsequently back-translated into English by an independent bilingual translator who had not participated in the forward translation. The original and back-translated English versions were then compared, and any discrepancies identified were reviewed and resolved by consensus by a bilingual committee comprising the translators and members of the research team. Finally, the Lithuanian version was pilot-tested with 13 Lithuanian-speaking adults from the target population to assess the clarity of the items. Participant feedback was reviewed, and no revisions were deemed necessary. The final Lithuanian version is presented in Appendix A Table A1.

### 2.3. Ethics Approval and Consent to Participate

The study was conducted in accordance with the ethical and legal principles of the Declaration of Helsinki. The research instruments and study procedures were reviewed and approved by the Biomedical Research Scientific-Ethics Committee of the Health Research and Innovation Science Centre, Faculty of Health Sciences, Klaipeda University (approval No. STIMC-BTMEK-05, 3 March 2025). Electronic informed consent was obtained from all participants included in the final sample.

### 2.4. Measures

PsyCap was assessed using the Lithuanian adaptation of the revised Compound Psychological Capital Scale (CPC-12R) (Dudasova et al., 2021). The original CPC-12R consists of 12 items rated on a 6-point Likert scale ranging from 1 (strongly disagree) to 6 (strongly agree). The scale measures PsyCap through four theoretically defined dimensions: optimism, hope, self-efficacy, and resilience, with each dimension represented by three items. A total score was calculated by averaging all items, with higher scores indicating higher levels of PsyCap.

Self-esteem was assessed using the Rosenberg Self-Esteem Scale (RSES) (Rosenberg, 1979). This 10-item self-report tool is designed to assess overall self-worth and general self-acceptance. The scale comprises both positively worded items (e.g., “On the whole, I am satisfied with myself”) and negatively worded items (e.g., “At times I think I am no good at all”). Participants responded to each item using a 4-point Likert scale, ranging from 1 (strongly disagree) to 4 (strongly agree). Negatively worded items were reverse-scored, and a total self-esteem score was calculated by summing the responses, resulting in scores ranging from 10 to 40. Higher scores denote greater self-esteem. The RSES has demonstrated cross-cultural factorial validity and has been used in large multinational samples, including Lithuania (Schmitt & Allik, 2005). In the current study, the RSES demonstrated good composite reliability (McDonald’s ω = 0.89, 95% CI = 0.88–0.90).

Life satisfaction was evaluated using the Satisfaction With Life Scale (SWLS) (Diener et al., 1985). This 5-item self-report instrument is intended to measure individuals’ overall cognitive assessment of their life satisfaction. The scale comprises items that reflect general life satisfaction, such as, “If I could live my life over, I would change almost nothing”. Participants rated each item on a 7-point Likert scale, from 1 (strongly disagree) to 7 (strongly agree). A mean life satisfaction score was derived by averaging the responses, with higher scores signifying greater life satisfaction. The SWLS has shown robust psychometric properties and evidence of measurement invariance across various national, linguistic, gender, and age groups, including Lithuania (Swami et al., 2025). In the current study, the SWLS demonstrated good composite reliability (McDonald’s ω = 0.89, 95% CI = 0.88–0.90).

Psychological distress was evaluated using the Kessler Psychological Distress Scale (K10) (Kessler et al., 2002). This 10-item self-report measure is designed to assess general psychological distress experienced over the preceding four weeks. The instrument is intended to identify symptoms associated with anxiety and depression. The scale comprises items indicative of emotional distress, for example, “During the past 4 weeks, about how often did you feel so nervous that nothing could calm you down?” Participants provided responses to each item on a 5-point Likert scale, ranging from 1 (none of the time) to 5 (all of the time). A mean psychological distress score was derived by averaging the item responses, with higher scores indicating greater psychological distress. The Lithuanian version of the K10 has demonstrated a one-factor structure and satisfactory model fit within a Lithuanian sample (Bacevičienė & Reviakaitė, 2025). In the current study, the K10 demonstrated excellent composite reliability (McDonald’s ω = 0.93, 95% CI = 0.92–0.94).

General mental health difficulties were assessed using the 12-item General Health Questionnaire (GHQ-12) (Goldberg et al., 1997). This 12-item self-report instrument is widely used to assess general mental health and psychological strain experienced during the past 3–4 weeks. The scale includes items related to emotional state, stress, anxiety, depressive symptoms, social functioning, and loss of confidence. It comprises both negatively worded items reflecting mental health difficulties, such as difficulty sleeping because of worries and feeling unhappy or depressed, and positively worded items reflecting psychological functioning, such as being able to concentrate and feeling capable of making decisions. Participants responded to each item using a 4-point response scale indicating the frequency of each experience. In the present study, items were scored so that higher GHQ-12 scores reflected poorer mental health and more mental health difficulties. A mean GHQ-12 score was calculated by averaging the item responses. The psychometric properties of the Lithuanian version of the GHQ-12 have been documented in previous studies (Malinauskiene et al., 2011). In the current study, the GHQ-12 demonstrated good composite reliability (McDonald’s ω = 0.89, 95% CI = 0.88–0.90).

Nature exposure was assessed using the Nature Exposure Scale (NES) (Kamitsis & Francis, 2013). This 4-item self-report measure is designed to assess individuals’ subjective exposure to natural environments in both everyday and non-everyday settings, as well as the extent to which they notice natural surroundings. The scale includes items assessing the amount of nature in participants’ everyday environment, the extent to which they notice nature in everyday life, the frequency of exposure to nature-rich environments outside their everyday environment, and the extent to which they notice nature in such settings. Participants responded to each item using a 5-point Likert-type scale, with higher scores indicating greater self-reported exposure to natural environments and greater awareness of surrounding nature. A mean NES score was calculated by averaging the item responses. Previous studies have supported the unidimensional factor structure and acceptable internal consistency of the NES, including its Lithuanian version (Matukynienė et al., 2021). In the current study, the NES demonstrated acceptable composite reliability (McDonald’s ω = 0.76, 95% CI = 0.74–0.79).

Physical activity in nature was assessed using the Physical Activity in Nature measure (PAN), which consisted of a season-specific item adapted from a Lithuanian national survey measure of physical activity (Klumbiene et al., 2015) and previously applied to assess physical activity in natural environments among Lithuanian adults (Jankauskiene & Baceviciene, 2025). Participants were asked how often they engaged in physical activities such as exercising, walking, cycling, or performing physically demanding tasks in natural settings, for example, forests or parks. Responses were provided on a 6-point Likert-type scale ranging from 1 (never or very rarely) to 6 (every day), with intermediate response options indicating different frequencies of participation. To account for seasonal variation, participants rated their physical activity in nature separately for spring, summer, autumn, and winter. A mean PAN score was calculated by averaging responses across the four seasons, with higher scores indicating more frequent physical activity in natural environments. In the current study, the PAN demonstrated excellent composite reliability (McDonald’s ω = 0.93, 95% CI = 0.93–0.94).

Leisure-time physical activity was assessed using the Leisure-Time Exercise Questionnaire (LTEQ) (Godin, 2011). This self-report instrument estimates participants’ weekly engagement in leisure-time physical activity, regardless of whether the activity is performed in natural environments. Participants reported how often they engaged in mild, moderate, and strenuous physical activities lasting at least 15 min during a typical week. Examples of mild activities include light walking and yoga, moderate activities include easy cycling, tennis, volleyball, badminton, and easy swimming, and strenuous activities include running and other vigorous exercise. Following the standard scoring procedure, the frequency of mild activity was multiplied by 3, moderate activity by 5, and strenuous activity by 9. The weighted scores for each intensity level were then summed to obtain a total leisure-time physical activity score. Higher scores indicated higher levels of self-reported leisure-time physical activity.

Sociodemographic data included gender, age, educational attainment, marital status, place of residence, and perceived financial security. Marital status was classified as single, single but in a committed relationship, or married/living with a partner. Place of residence was categorized as rural/suburban or urban. Perceived financial security was assessed relative to peers and categorized as less financially secure, financially similar, or more financially secure.

### 2.5. Statistical Analysis

All analyses were conducted using R v.4.6.0 (R Foundation for Statistical Computing, Vienna, Austria), SPSS v.31 (IBM Corp., Armonk, NY, USA) and JASP (version 0.97 (JASP Team, University of Amsterdam, Amsterdam, The Netherlands). Descriptive statistics, exploratory factor analysis (EFA), Pearson correlation analyses, composite reliability estimates, and hierarchical regression analyses were performed in JASP and SPSS. Confirmatory factor analyses (CFAs) and measurement invariance testing were conducted in R using the lavaan package, whereas multivariate normality was assessed using Mardia’s tests implemented in the MVN package.

The total sample was randomly split into two subsamples. Sample adequacy for factor analysis was supported by the large participant-to-item ratio, which exceeds conventional recommendations of at least 10 participants per item (Worthington & Whittaker, 2006). In the first subsample, EFA was conducted using Principal Axis Factoring with Promax rotation to explore the underlying factor structure of the CPC-12R (Costello & Osborne, 2005). Sampling adequacy was evaluated using the Kaiser–Meyer–Olkin (KMO) measure, and Bartlett’s test of sphericity was used to assess the suitability of the data for factor analysis.

The factor structure of the Lithuanian version of the CPC-12R was evaluated using confirmatory factor analysis. Competing 4- and 2-factor first-order and second-order models were examined in a randomly selected half of the sample (*n* = 627), and the best-fitting model was subsequently cross-validated in the remaining half of the sample. Multivariate normality was assessed using Mardia’s tests of multivariate skewness and kurtosis (Korkmaz et al., 2014; Sánchez, 1982). Because significant departures from multivariate normality were observed, robust maximum likelihood estimation (MLR) was employed. Model fit was evaluated using robust Comparative Fit Index (CFI), robust Tucker–Lewis Index (TLI), robust Root Mean Square Error of Approximation (RMSEA), and Standardized Root Mean Square Residual (SRMR). Following conventional recommendations, CFI and TLI values ≥ 0.90 were considered acceptable and ≥0.95 indicative of good fit, RMSEA values ≤ 0.08 acceptable and ≤0.06 good, and SRMR values ≤ 0.08 indicative of good fit (Hu & Bentler, 1999).

Measurement invariance of the final second-order CPC-12R model was examined across gender, education and place-of-residence groups. Configural, metric, scalar, and strict invariance models were estimated using robust maximum likelihood estimation. Invariance was evaluated using changes in model fit indices, with decreases in CFI smaller than 0.010 and increases in RMSEA smaller than 0.015 indicating support for invariance (Chen, 2007).

Composite reliability was assessed using McDonald’s ω coefficients with 95% confidence intervals (Dunn et al., 2014). To assess the potential influence of common method variance (CMV), a post hoc single-factor confirmatory factor analysis (CFA) including all item-level self-report measures used in the validity analyses was conducted. Convergent and criterion-related validity were examined through Pearson correlations between CPC-12R, self-esteem, life satisfaction, psychological distress, mental health, nature exposure, physical activity in nature, and leisure-time physical activity. Correlation effect sizes were interpreted according to Cohen (1988), with coefficients of r = 0.10, 0.30, and 0.50 representing small, medium, and large effects, respectively (Cohen, 1988).

To examine the incremental validity of CPC-12R, a hierarchical linear regression analysis was conducted with poor mental health (GHQ-12 total score) as the dependent variable. In the first step, psychological distress, life satisfaction, and self-esteem were entered as predictors. In the second step, CPC-12R was added to determine whether it explained additional variance in mental health beyond these established psychological correlates. Incremental validity was evaluated using the change in explained variance (ΔR^2^).

Finally, a second hierarchical regression analysis was performed to investigate predictors of CPC-12R. In the first step, sociodemographic variables (gender, age, educational attainment, marital status, place of residence, and perceived financial security) were entered. In the second step, nature exposure, physical activity in nature, and leisure-time physical activity were added to assess their additional contribution to explaining variance CPC-12R. Changes in explained variance (ΔR^2^) were used to evaluate the incremental predictive value of the nature-related variables. Statistical significance was set at *p* < 0.05 for all analyses.

## 3. Results

The sample comprised 1255 adults, with women representing the majority of participants. Most respondents had higher education and were married or living with a partner. Participants were predominantly urban or suburban residents. The majority perceived their financial situation as average compared with others, while smaller proportions reported feeling either less or more financially secure. Table 1 presents the detailed sociodemographic characteristics of the study sample.

**Table 1:** Sociodemographic characteristics of the study participants (*n* = 1255).

Characteristics		*n*	%
Gender	men	447	35.6
	women	808	64.4
Education	secondary	104	8.3
	apprenticeship	87	6.9
	in full-time studies	213	17.0
	higher	851	67.80
Marital status	single	322	25.7
	single, but in a committed relationship	172	13.7
	married, living with a partner	761	60.6
Place of residence	rural/suburban	379	30.2
	urban	876	69.8
Perceived financial security	less secure than others	198	15.8
	average, like most	816	65.0
	more secure than others	241	19.2

Next, the total sample (*n* = 1255) was randomly divided into two equal subsamples for exploratory and confirmatory analyses (*n* = 628 for EFA and *n* = 627 for CFA). To evaluate whether the subsamples were comparable, differences in age and gender distribution were examined. An independent-samples t-test indicated no significant age difference between the EFA subsample (M = 39.50 years, SD = 13.87) and the CFA subsample (M = 39.52 years, SD = 13.83), t(1252) = 0.04, *p* = 0.972, d = 0.002. Likewise, the gender distribution did not differ significantly between subsamples, χ^2^(1, *n* = 1255) = 0.09, *p* = 0.768. These findings indicate that the EFA and CFA subsamples were comparable in terms of age and gender composition.

Item-level descriptive statistics are presented in the Appendix A Table A2. Mean item scores ranged from 4.17 to 4.93, with generally moderate negative skewness and no substantial distributional concerns.

Exploratory factor analysis was conducted on a random half of the sample (*n* = 628) using Principal Axis Factoring with Promax rotation. Sampling adequacy was excellent (KMO = 0.931), and Bartlett’s test of sphericity was significant, χ^2^(66) = 4723.36, *p* < 0.001, indicating suitability for factor analysis. Two factors with eigenvalues greater than one were extracted, accounting for 65.9% of the total variance (Table 2). The first factor (eigenvalue = 6.77) explained 56.4% of the variance and comprised items reflecting hope, self-efficacy, and resilience. The second factor (eigenvalue = 1.14) explained 9.5% of the variance and consisted exclusively of the three optimism items. All retained items demonstrated substantial loadings on their primary factor (0.48–0.88) and satisfactory communalities (0.39–0.69). The correlation between Factor 1 and Factor 2 was r = 0.723.

**Figure 2:**
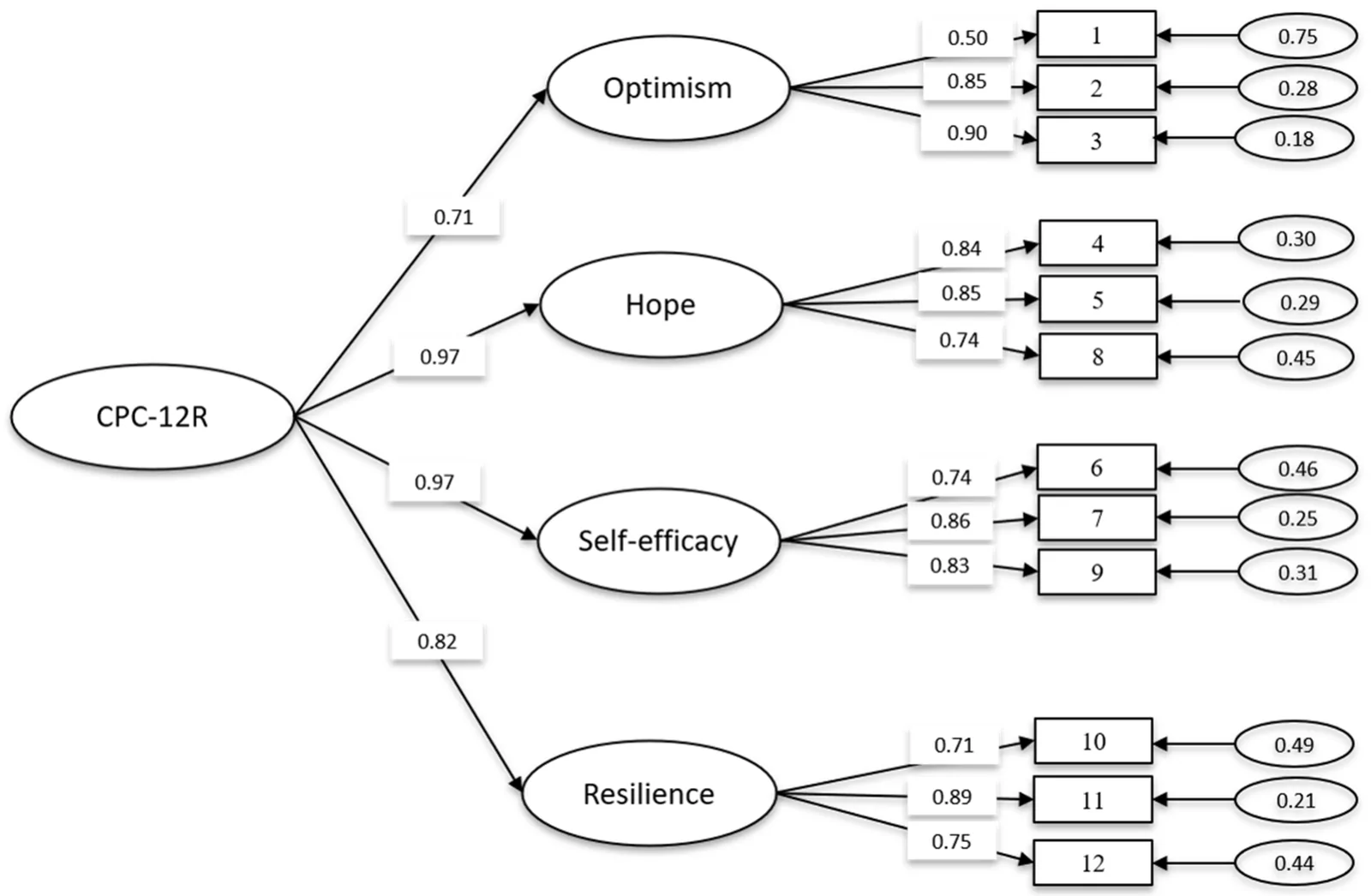
Second-order confirmatory factor model of the Compound Psychological Capital Scale (*n* = 627). Note. CPC-12R = the revised Compound Psychological Capital Scale; large ellipses denote latent variables, rectangles denote observed items, and small ellipses denote standardized residual variances. Values on the arrows are standardized factor loadings from the higher-order factor to the first-order factors and from the first-order factors to the observed items.

Confirmatory factor analysis was conducted in the validation subsample (*n* = 627) using robust maximum likelihood estimation (MLR). Three theoretically plausible models were examined: the original four-factor first-order model, a two-factor model separating optimism from the remaining psychological capital dimensions, and a second-order model specifying optimism, hope, self-efficacy, and resilience as first-order factors reflected by a higher-order psychological capital construct. Although all models demonstrated acceptable fit, the second-order model showed the best overall fit to the data (robust CFI = 0.963, robust TLI = 0.952, robust RMSEA = 0.074, robust SRMR = 0.042) and was therefore retained as the final measurement model. As shown in Figure 2, standardized factor loadings of the items on their respective first-order factors ranged from 0.50 to 0.90, while standardized loadings of the first-order factors on the higher-order PsyCap factor ranged from 0.71 to 0.97, indicating strong relationships between the observed indicators, first-order dimensions, and the higher-order construct.

Measurement invariance of the second-order CPC-12R model was examined across gender using multigroup confirmatory factor analysis with robust maximum likelihood estimation (MLR). The configural model demonstrated acceptable fit, supporting the equivalence of the basic factor structure across men and women (Table 3). Metric invariance was supported, as the change in comparative fit index did not exceed the recommended cutoff of 0.10. Scalar invariance was also supported (ΔCFI = 0.004), indicating that latent mean comparisons across gender are appropriate. Strict invariance was additionally supported (ΔCFI = 0.001), demonstrating equivalence of residual variances across groups. Measurement invariance across education groups was supported at the configural, metric, and scalar levels, whereas strict invariance was not supported. Across place-of-residence groups, configural, metric, scalar, and strict invariance were supported.

Following measurement invariance testing, total CPC-12R scores were compared across gender, education, and place-of-residence groups. No significant gender difference in PsyCap was observed, *t*(1253) = −0.236, *p* = 0.814, Cohen’s *d* = −0.014. Participants with higher education reported higher PsyCap scores than those with non-higher education (*M* = 4.646 vs. 4.387), *t*(1253) = −5.386, *p* < 0.001, Cohen’s *d* = −0.325. Urban participants also reported slightly higher PsyCap scores than rural/suburban participants (*M* = 4.592 vs. 4.494), *t*(1253) = −1.987, *p* = 0.047, Cohen’s *d* = −0.122.

Internal consistency estimates indicated satisfactory to excellent reliability for the CPC-12R scale and its dimensions. McDonald’s ω ranged from 0.79 (95% CI = 0.76–0.82) for Optimism to 0.85 (95% CI = 0.83–0.87) for Hope. Reliability estimates were 0.84 (95% CI = 0.82–0.86) for Self-Efficacy and 0.82 (95% CI = 0.80–0.84) for Resilience. The total 12-item CPC-12R scale demonstrated excellent internal consistency (McDonald’s ω = 0.93, 95% CI = 0.92–0.93).

A post hoc single-factor CFA was conducted to assess the potential influence of common method variance. The one-factor model demonstrated poor fit (CFI = 0.439, TLI = 0.423, RMSEA = 0.103, SRMR = 0.117), indicating that a single common factor could not adequately explain the covariance among the self-report measures. Therefore, common method variance is unlikely to represent the primary explanation for the observed associations.

Table 4 summarizes correlations between CPC-12R and supporting study measures. As expected, PsyCap demonstrated strong positive associations with self-esteem and life satisfaction and strong negative associations with psychological distress and poorer mental health, providing evidence of convergent validity. These findings indicate that individuals with higher levels of PsyCap tend to report greater psychological well-being, more positive self-evaluations, and fewer symptoms of distress and mental health difficulties. PsyCap was also positively associated with nature exposure, physical activity in nature, and leisure-time exercise. The strongest associations were observed for nature exposure and physical activity in nature, whereas the relationship with overall leisure-time exercise was comparatively weaker. These findings support the convergent and criterion validity of the scale and suggest that PsyCap is linked not only to well-being but also to engagement with natural environments and health-promoting lifestyle behaviours.

To examine the incremental validity of the revised CPC-12R, a hierarchical regression analysis was conducted to predict mental health (Table 5). In Step 1, self-esteem, life satisfaction, and psychological distress explained 62% of the variance in mental health. Higher psychological distress was associated with poorer mental health, whereas higher self-esteem and life satisfaction were associated with better mental health. In Step 2, PsyCap was added to the model. The inclusion of PsyCap resulted in a significant increase in explained variance (ΔR^2^ = 0.014, *p* < 0.001), increasing the total explained variance to 64%. PsyCap remained a significant predictor of mental health after controlling for self-esteem, life satisfaction, and psychological distress, providing evidence of the incremental validity of the scale. In the final model, self-esteem, psychological distress, and PsyCap remained significant predictors, whereas life satisfaction was no longer a significant unique predictor of mental health. Multicollinearity was not a concern, as VIF values ranged from 1.53 to 2.23.

A hierarchical regression analysis was conducted to identify sociodemographic and nature-related predictors of PsyCap (Table 6). In Step 1, sociodemographic variables explained 10.6% of the variance in PsyCap. Higher educational attainment, greater perceived financial security, being married/cohabiting, and living in an urban area were associated with higher PsyCap. In Step 2, nature exposure, physical activity in nature, and leisure-time exercise were added to the model, resulting in a significant increase in explained variance (ΔR^2^ = 0.091, *p* < 0.001), with the final model accounting for 19.7% of the variance in PsyCap. Greater nature exposure and more frequent physical activity in nature were significant positive predictors of PsyCap, whereas leisure-time exercise was not. In the final model, higher education, greater financial security, and urban residence also remained significant predictors of PsyCap, while age showed a small negative association. In addition, participants who were married or cohabiting reported higher levels of PsyCap than single participants, whereas being in a committed relationship without cohabitation was not associated with PsyCap. Examination of multicollinearity diagnostics indicated no evidence of problematic multicollinearity, with VIF values ranging from 1.00 to 1.20.

## 4. Discussion

In the present study, we sought to examine whether the Lithuanian translation of the CPC-12R represents a valid and reliable measure of PsyCap and whether it is positively related to nature exposure, physical activity in nature, and overall physical activity. The findings generally supported our expectations. Consistent with our hypothesis and with the original validation study by (Dudasova et al., 2021), CFA supported the theoretically expected second-order model of the CPC-12R, in which optimism, hope, self-efficacy, and resilience function as first-order factors reflecting a higher-order PsyCap construct. The second-order model demonstrated good fit across conventional indices, and standardized factor loadings were strong throughout, indicating that the items adequately represent their respective first-order dimensions and that these dimensions are coherent manifestations of a broader PsyCap construct. These findings are consistent with prior national language adaptations of the CPC-12R in Czech, Slovak, German, English, Japanese, and Arabic samples (Ikeda et al., 2022; Kačmár et al., 2022; Lorenz et al., 2022; Mansouri & Lorenz, 2025; Prochazka et al., 2025), supporting the cross-cultural generalizability of the scale’s factor structure and extending this evidence to a Lithuanian-speaking adult population.

### 4.1. Psychometric Properties of Lithuanian Translation of CPC-R12

Exploratory factor analysis of the first subsample yielded a two-factor solution, with optimism items forming a distinct factor separate from hope, self-efficacy, and resilience items. Although this pattern appears inconsistent with the theoretical four-factor structure of PsyCap, it is not entirely unprecedented in previous research. Optimism is generally defined as a tendency to expect positive outcomes in the future. This distinguishes it from hope, which emphasizes goal-directed motivation and the ability to identify pathways to achieve goals, and from self-efficacy and resilience, which reflect confidence in one’s abilities and the capacity to adapt to and recover from adversity (Luthans & Youssef-Morgan, 2017). Despite their conceptual distinctiveness, hope, self-efficacy, and resilience share a strong agentic and adaptive orientation, collectively reflecting individuals’ perceived capacity to identify pathways towards their goals, act effectively, and persist or recover when facing difficulties. In the present Lithuanian sample, these three dimensions may therefore have operated as a highly integrated coping resource at the item level. Given these conceptual distinctions, the emergence of a separate optimism factor may reflect the unique nature of this PsyCap component. Notably, previous national language validation studies of the original CPC-12R have relied exclusively on CFA, whereas the present study is among the first to examine the scale using EFA. Consequently, the observed two-factor solution may reflect characteristics of the revised CPC-12R items, the Lithuanian cultural context, or a combination of both, and therefore warrants further investigation. However, CFA supported the theoretically proposed four-factor second-order structure and demonstrated a better fit than competing models. Nevertheless, the EFA findings indicate that the theoretical differentiation among the four PsyCap dimensions was not fully reproduced at the item level in the Lithuanian sample. Specifically, hope, self-efficacy, and resilience appeared to reflect a broadly integrated agentic and adaptive resource, whereas optimism emerged as a more distinct cognitive component. Although the CFA findings support the higher-order conceptualisation of PsyCap, they do not entirely resolve the conceptual ambiguity revealed by the EFA regarding the empirical distinctiveness of its first-order dimensions. Thus, the findings provide qualified rather than unequivocal support for the construct validity of the Lithuanian CPC-12R, particularly when separate subscale scores are interpreted. Further research is needed to determine whether this pattern is specific to the present sample or reflects a more general feature of PsyCap in the Lithuanian context. Taken together, the EFA and CFA findings suggest that, although optimism may emerge as a distinguishable component at the item level, it remains consistent with the higher-order conceptualization of PsyCap.

Reliability analyses further supported the psychometric adequacy of the Lithuanian CPC-12R. Internal consistency estimates were satisfactory to good for all four subscales and excellent for the total scale, extending the reliability evidence reported in previous validation studies (Hussain & Habib, 2026; Ikeda et al., 2022; Kačmár et al., 2022; Lorenz et al., 2022; Mansouri & Lorenz, 2025). Nevertheless, the high reliability of the total score should be interpreted cautiously, as it may partly reflect the substantial shared variance among the PsyCap dimensions and should not, by itself, be regarded as evidence of a clearly differentiated four-factor structure.

The second-order CPC-12R model demonstrated all levels of measurement invariance across gender, indicating that observed score differences or similarities can be meaningfully interpreted. Evidence for gender measurement invariance of the CPC-12R remains extremely limited, with only one national validation study (the Japanese version) demonstrating full scalar invariance (Ikeda et al., 2022), while other studies have either not examined invariance or reported only partial evidence (Mansouri & Lorenz, 2025). As such, the present study extends the existing literature by providing one of the few demonstrations of full gender invariance of the CPC-12R, thereby strengthening confidence in its use for gender comparisons in future PsyCap research. Consistent with findings from the Japanese validation study (Ikeda et al., 2022), no significant gender differences in PsyCap were observed. This finding is consistent with evidence from studies using the Psychological Capital Questionnaire (Luthans et al., 2007) which indicates that gender differences in PsyCap are typically small or non-significant (Newman et al., 2014).

Evidence of convergent validity was demonstrated through positive associations between PsyCap and self-esteem and life satisfaction, and negative associations with psychological distress and poorer mental health. These patterns are theoretically expected: individuals with greater PsyCap are better positioned to appraise themselves positively, maintain positive expectations, regulate emotional responses to adversity, and sustain a sense of life satisfaction. Similar patterns of correlations were observed in other validation studies (Ikeda et al., 2022; Kačmár et al., 2022; Lorenz et al., 2022; Mansouri & Lorenz, 2025; Prochazka et al., 2025).

PsyCap demonstrated statistically significant but limited incremental validity in relation to mental health difficulties beyond self-esteem, life satisfaction, and psychological distress. Its inclusion increased the explained variance by 1.4%, indicating that its unique contribution was small despite being statistically significant. To our knowledge, neither the original CPC-12R study nor subsequent national-language validation studies have examined the instrument’s incremental validity (Dudasova et al., 2021; Ikeda et al., 2022; Kačmár et al., 2022; Lorenz et al., 2022; Mansouri & Lorenz, 2025; Prochazka et al., 2025). The modest increment observed in the present study may partly reflect the substantial conceptual and empirical overlap of PsyCap with self-esteem and life satisfaction. In addition, the variables entered in the first step already explained 62.3% of the variance in GHQ-12 scores, leaving relatively little unexplained variance for PsyCap to account for. In particular, psychological distress and mental health difficulties represent closely related constructs, making this a stringent test of incremental validity. Therefore, the findings provide modest rather than strong evidence that PsyCap captures unique aspects of psychological functioning beyond the established correlates included in the model. Future studies should examine its incremental validity in relation to a broader range of less conceptually overlapping outcomes and assess whether its unique contribution is replicated in longitudinal data.

### 4.2. Nature Exposure, Physical Activity, and Psychological Capital

A central and novel contribution of the present study is the examination of nature exposure and physical activity as correlates and predictors of PsyCap. In the present study PsyCap was positively associated with nature exposure, physical activity in nature, and leisure-time exercise, with the strongest associations observed for the nature-based variables. In the hierarchical regression, greater nature exposure and more frequent physical activity in nature emerged as significant positive predictors of PsyCap after controlling for sociodemographic variables, whereas general leisure-time exercise was not a significant predictor. This differential pattern is theoretically informative and warrants discussion.

The finding that nature exposure independently predicted PsyCap is consistent with the broader literature linking natural environments to positive psychological outcomes (Hartig et al., 2014; Houlden et al., 2018). From the perspective of Attention Restoration Theory, natural environments facilitate the recovery of directed attentional resources, thereby reducing mental fatigue and supporting more effective cognitive and emotional regulation (Kaplan, 1995). From the perspective of Stress Recovery Theory, exposure to natural settings elicits positive affective and physiological responses that reduce arousal and stress (Ulrich et al., 1991). Both mechanisms may plausibly contribute to the development of PsyCap dimensions such as resilience, which requires effective recovery from stress, and optimism, which is supported by positive affective states (Fredrickson, 2001). Furthermore, regular engagement with natural environments may cultivate mindful awareness and a sense of connection to something larger than oneself (Barragan-Jason et al., 2023; Lee et al., 2015; Swami et al., 2024), both of which have been linked to enhanced psychological well-being and to the positive cognitive and motivational processes underpinning PsyCap.

The independent predictive value of physical activity in nature, over and above general leisure-time physical activity, suggests that the relationship between physical activity and PsyCap may depend not only on how much activity individuals engage in but also on where that activity takes place. This finding is consistent with the “green exercise” literature, which proposes that natural environments provide psychological benefits that extend beyond those attributable to physical activity or nature exposure alone (Brito et al., 2022; Christiana et al., 2021; Coventry et al., 2021; Laezza et al., 2025; Peddie et al., 2024; Twohig-Bennett & Jones, 2018; Wicks et al., 2022). One possible explanation is offered by the affordance perspective, which suggests that natural environments provide opportunities for perception and action that differ from those available in urban or built settings (Araújo et al., 2019). By engaging with these nature-based affordances, individuals may experience a wider range of sensory, cognitive, and behavioural interactions, fostering deeper physical, psychological, and emotional engagement with their surroundings. Such experiences may promote positive emotions, attentional restoration, stress recovery, and perceptions of competence and mastery, all of which are theoretically relevant to the development of PsyCap (Araújo et al., 2019). For example, successfully engaging with the opportunities afforded by natural environments may strengthen self-efficacy, resilience, and optimism. In contrast, general leisure-time physical activity encompasses a broad range of activities and settings, many of which may not provide these additional psychological benefits. Thus, the absence of a significant association between general physical activity and PsyCap in the regression model suggests that the beneficial relationship between physical activity and PsyCap may be driven primarily by the natural context in which activity occurs rather than by activity frequency alone. Although our findings are consistent with previous research reporting positive associations between general physical activity and PsyCap (Huang et al., 2025), they extend the existing literature by demonstrating that physical activity in nature is a stronger and more robust correlate of PsyCap than general leisure-time physical activity.

### 4.3. Sociodemographic Correlates of Psychological Capital

Among the sociodemographic variables, greater perceived financial security, higher educational attainment, married or cohabiting status, and urban residence were significant positive correlates of PsyCap. The positive associations between socioeconomic indicators and PsyCap are consistent with previous research (Dóci et al., 2023; Moortel et al., 2024). Access to economic, cultural, and social resources may facilitate the development and maintenance of positive psychological resources such as hope, self-efficacy, resilience, and optimism. Similarly, the positive associations of urban residence status with PsyCap may reflect greater access to educational, occupational, and social resources, as well as higher levels of social support and psychological well-being. Together, these findings support idea that Bourdieu’s capital framework includes not only economic, cultural and social capital but also PsyCap (Moortel et al., 2024). In contrast, the positive association between marital status and PsyCap may reflect the benefits of close interpersonal relationships. Marriage and cohabitation are often associated with greater social support, emotional stability, life satisfaction, and psychological well-being (Hsu & Barrett, 2020; Spahni & Perrig-Chiello, 2018), factors that may facilitate the development and maintenance of PsyCap.

### 4.4. Practical Implications

The present findings may have practical implications for workplace health promotion initiatives. Many organizations seek to enhance employee well-being and performance by encouraging physical activity, often through subsidized gym memberships, workplace fitness programmes, or partnerships with indoor sport and exercise facilities. In Lithuania, it is common for employers to provide employees with collective memberships to fitness clubs as part of workplace well-being initiatives. While such programmes may increase physical activity participation, the present findings suggest that the context in which physical activity occurs may also be important. Consequently, health promotion practitioners, community organizations, educational institutions, and employers may benefit from incorporating nature-based activities into existing well-being initiatives.

### 4.5. Limitations and Future Directions

Several limitations of the present study should be acknowledged. First, the cross-sectional design precludes causal inference, and the direction of associations between nature exposure, physical activity, and PsyCap cannot be established from the present data. Longitudinal and experimental designs are needed to determine whether increased nature engagement and physical activity in nature prospectively predict PsyCap development, or whether individuals with greater PsyCap are more likely to seek out natural environments. Second, the sample was recruited via non-probabilistic volunteer sampling, which may have introduced self-selection bias. The overrepresentation of women, individuals with higher education, and urban residents limits the generalizability of the findings to the broader Lithuanian adult population. Third, all variables were assessed using self-report measures within the same survey and at the same time point, which introduces the possibility of common method variance and social desirability bias and may have inflated some of the observed associations. Furthermore, physical activity in nature was assessed retrospectively across the four seasons, making these estimates susceptible to recall bias and inaccuracies in participants’ recollection of their season-specific activity. Future studies would benefit from incorporating objective measures of nature exposure and physical activity, such as GPS-based or ecological momentary assessment methods, alongside self-reported PsyCap, as well as temporally separating the assessment of predictor and outcome variables. Fourth, the present study examined nature exposure and physical activity in nature as predictors but did not assess potential psychological mediators, such as nature connectedness, mindfulness, body image or restorative experience, that may explain why nature engagement is associated with PsyCap. Examining such mechanisms would provide a more complete theoretical account of the nature–PsyCap relationship. Finally, although the CPC-12R demonstrated strong psychometric properties in the present general adult sample, further validation is warranted. Future research should examine its psychometric properties in specific Lithuanian populations, such as occupational and clinical groups, and assess test–retest reliability to provide additional evidence regarding the temporal stability of scores.

## 5. Conclusions

The present study provides support for the reliability and validity of the Lithuanian adaptation of the CPC-12R in a general adult sample. CFA supported the theoretically expected second-order model comprising four first-order dimensions, whereas EFA distinguished optimism from a broader factor encompassing hope, self-efficacy, and resilience. The scale demonstrated excellent internal consistency for the total score, satisfactory-to-good reliability for the subscales, strict measurement invariance across gender and place-of-residence groups. It also showed convergent validity and statistically significant, although modest, incremental validity in relation to mental health difficulties. Taken together, the findings support the use of the total CPC-12R score, while suggesting that the separate subscale scores should be interpreted with caution pending further validation. Nature exposure and physical activity in natural environments were independently positively associated with PsyCap after accounting for sociodemographic characteristics, whereas general leisure-time exercise was not. These cross-sectional findings identify engagement with natural environments as a potentially relevant behavioural correlate of PsyCap.

## Figures and Tables

**Table 2:** Exploratory factor analysis of the CPC-12R: pattern matrix and communalities (*n* = 628).

Item	Factor 1	Factor 2	h^2^
CPC-12R-1	−0.03	0.65	0.39
CPC-12R-2	−0.15	0.88	0.60
CPC-12R-3	0.11	0.74	0.67
CPC-12R-4	0.58	0.28	0.66
CPC-12R-5	0.61	0.24	0.64
CPC-12R-6	0.48	0.30	0.53
CPC-12R-7	0.77	0.08	0.69
CPC-12R-8	0.56	0.25	0.57
CPC-12R-9	0.88	−0.08	0.68
CPC-12R-10	0.78	−0.06	0.55
CPC-12R-11	0.87	−0.11	0.63
CPC-12R-12	0.75	−0.08	0.48

Note. CPC-12R = the revised Compound Psychological Capital Scale; h^2^ = communality. Extraction method: principal axis factoring; rotation method: Promax with Kaiser normalization.

**Table 3:** Measurement invariance models of the revised Compound Psychological Capital Scale across gender, education and place of residence groups (*n* = 1255).

Model	Robust CFI	Robust TLI	Robust RMSEA	SRMR	∆CFI	∆RMSEA	∆SRMR
Gender group invariance (men vs. women)							
Configural	0.955	0.940	0.082	0.038	–	–	–
Metric	0.953	0.945	0.079	0.051	0.002	0.003	0.013
Scalar	0.949	0.943	0.080	0.053	0.004	0.001	0.002
Strict	0.950	0.949	0.076	0.053	0.001	0.004	0.000
Education group invariance (non-higher vs. higher)							
Configural	0.958	0.942	0.080	0.036	–	–	–
Metric	0.957	0.945	0.078	0.042	−0.001	−0.002	0.006
Scalar	0.955	0.947	0.077	0.043	−0.002	−0.001	0.001
Strict	0.943	0.939	0.083	0.046	−0.012	0.006	0.003
Place of residence group invariance (urban vs. rural/suburban)							
Configural	0.955	0.940	0.082	0.038	–	–	–
Metric	0.955	0.947	0.077	0.042	0.000	−0.005	0.004
Scalar	0.956	0.950	0.075	0.043	0.001	−0.002	0.001
Strict	0.956	0.955	0.071	0.043	0.000	−0.004	0.000

Note. CFI = comparative fit index; RMSEA = Steiger-Lind root mean square error of approximation; SRMR = standardized root mean square residual; ∆ = change in model fit indices.

**Table 4:** Correlations between the revised Compound Psychological Capital Scale and supporting study measures (*n* = 1255).

Measures	PC	SE	LS	PD	MH	NE	PAN	LTE
Psychological capital (PC)	1.00							
Self-esteem (SE)	0.64 **	1.00						
Life satisfaction (LS)	0.54 **	0.58 **	1.00					
Psychological distress (PD)	−0.43 **	−0.57 **	−0.45 **	1.00				
Mental health (MH)	−0.52 **	−0.60 **	−0.46 **	0.76 **	1.00			
Nature exposure (NE)	0.29 **	0.31 **	0.35 **	−0.26 **	−0.25 **	1.00		
Physical activity in nature (PAN)	0.27 **	0.22 **	0.22 **	−0.15 **	−0.20 **	0.30 **	1.00	
Leisure-time exercise (LTE)	0.14 **	0.07 *	0.11 **	−0.01	−0.09 **	0.14 **	0.42 **	1.00

Note. * *p* < 0.05, ** *p* < 0.001.

**Table 5:** Incremental validity of the revised Compound Psychological Capital Scale when predicting mental health (*n* = 1255).

Measures	B	SE	β	t	*p*
Step 1					
Self-esteem	−0.20	0.022	−0.21	−8.96	<0.001
Life satisfaction	−0.03	0.009	−0.06	−2.92	0.004
Psychological distress	0.40	0.014	0.62	28.61	<0.001
Model summary: R = 0.789, R^2^ = 0.623, F = 689.18, *p* < 0.001					
Step 2					
Self-esteem	−0.13	0.024	−0.14	−5.30	<0.001
Life satisfaction	−0.01	0.010	−0.02	−1.02	0.309
Psychological distress	0.39	0.014	0.61	28.77	<0.001
Psychological capital	−0.10	0.014	−0.16	−6.93	<0.001
Model summary: R = 0.798, R^2^ = 0.637, ΔR^2^ = 0.014, F = 548.27, *p* < 0.001					

Note. B = unstandardized regression coefficient, β = standardized regression coefficient, SE = standard error.

**Table 6:** Hierarchical regression predicting psychological capital by sociodemographic characteristics, nature exposure and physical activity (*n* = 1255).

Measures	B	SE	β	t	*p*
**Step 1**					
Gender: women (ref. men)	0.00	0.046	–	0.09	0.925
Age	0.00	0.002	0.00	0.05	0.964
Education	0.07	0.018	0.11	3.80	<0.001
Marital status: in a committed relationship (ref. single)	0.02	0.072	–	0.23	0.816
Marital status: married/cohabiting (ref. single)	0.11	0.056	–	2.05	0.041
Urban place of residence (ref. rural/suburb)	0.11	0.047	–	2.36	0.019
Perceived financial security	0.36	0.037	0.26	9.61	<0.001
Model summary: R = 0.326, R^2^ = 0.106, F = 21.11, *p* < 0.001					
**Step 2**					
Gender: women (ref. men)	−0.04	0.046	–	−0.78	0.435
Age	−0.01	0.002	−0.08	−2.62	0.009
Education	0.09	0.017	0.14	4.93	<0.001
Marital status: in a committed relationship (ref. single)	0.00	0.068	–	0.01	0.995
Marital status: married/cohabiting (ref. single)	0.12	0.053	–	2.22	0.027
Urban place of residence (ref. rural/suburban)	0.13	0.045	–	2.88	0.004
Perceived financial security	0.28	0.036	0.20	7.67	<0.001
Nature exposure	0.20	0.027	0.21	7.42	<0.001
Physical activity in nature	0.10	0.017	0.17	5.68	<0.001
Leisure-time exercise	0.00	0.00	0.03	1.17	0.244
Model summary: R = 0.444, R^2^ = 0.197, ΔR^2^ = 0.091, F = 30.52, *p* < 0.001					

Note. B = unstandardized regression coefficient, β = standardised regression coefficient, SE = standard error.

## Data Availability

The data presented in this study are available from the corresponding author upon reasonable request. The dataset forms part of a larger ongoing research project, and public deposition has been deferred until the primary analyses from the project have been completed.

## Appendix A

**Table A1:** Original English and Lithuanian Adapted Versions of the Revised Compound Psychological Capital Scale (CPC-12R).

CPC-12R in English	CPC-12R in Lithuanian
1 = strongly disagree, 2 = disagree, 3 = slightly disagree, 4 = slightly agree, 5 = agree, 6 = strongly agree	1 = visiškai nesutinku, 2 = nesutinku, 3 = šiek tiek nesutinku, 4 = šiek tiek sutinku, 5 = sutinku, 6 = visiškai sutinku
1. I am looking forward to the life ahead of me.	1. Žvelgiu į gyvenimą, kuris manęs laukia ateityje.
2. Overall, I expect more good things to happen to me than bad.	2. Apskritai tikiuosi, kad man nutiks daugiau gerų dalykų nei blogų.
3. The future holds a lot of good in store for me.	3. Ateityje manęs laukia daug gero.
4. If I should find myself in a jam, I could think of many ways to get out of it.	4. Jei atsidurčiau keblioje situacijoje, galėčiau sugalvoti daug būdų, kaip iš jos išsisukti.
5. I can think of many ways to reach my current goals.	5. Galiu sugalvoti daug būdų, kaip pasiekti savo esamus tikslus.
6. Right now, I see myself as being pretty successful.	6. Šiuo metu save laikau gana sėkmingu (-a).
7. I am confident that I could deal efficiently with unexpected events.	7. Esu įsitikinęs (-usi), kad galėčiau efektyviai susidoroti su netikėtais įvykiais.
8. I can solve most problems if I invest the necessary effort.	8. Galiu išspręsti daugumą problemų, jei įdėsiu reikiamų pastangų.
9. I can remain calm when facing difficulties because I can rely on my coping abilities.	9. Kai susiduriu su sunkumais, galiu išlikti ramus (-i), nes pasitikiu savo gebėjimais juos įveikti.
10. I consider myself a person who can withstand a lot.	10. Laikau save žmogumi, kuris gali daug ką ištverti.
11. Failure does not discourage me.	11. Nesėkmės manęs neatbaido.
12. I tend to bounce back quickly after serious life difficulties.	12. Esu linkęs (-usi) greitai atsigauti po rimtų gyvenimo sunkumų.

Note. The original English version of the CPC-12R was developed by Dudasova et al. (2021). The Lithuanian adapted version was prepared for the present study.

**Table A2:** Descriptive statistics for individual CPC-12R items.

Item	Mean	SD	Skewness	Kurtosis	Range
CPC-12R-1	4.60	1.16	−1.11	1.25	1–6
CPC-12R-2	4.93	1.09	−1.29	1.75	1–6
CPC-12R-3	4.74	1.09	−0.91	0.65	1–6
CPC-12R-4	4.62	1.01	−0.83	1.10	1–6
CPC-12R-5	4.49	1.04	−0.70	0.62	1–6
CPC-12R-6	4.36	1.13	−0.73	0.39	1–6
CPC-12R-7	4.38	1.05	−0.68	0.57	1–6
CPC-12R-8	4.85	0.98	−0.95	1.25	1–6
CPC-12R-9	4.36	1.09	−0.69	0.29	1–6
CPC-12R-10	4.86	1.06	−1.12	1.33	1–6
CPC-12R-11	4.40	1.16	−0.67	0.09	1–6
CPC-12R-12	4.17	1.20	−0.52	−0.27	1–6

Note. CPC-12R = revised Compound Psychological Capital Scale; SD = standard deviation.

## References

[B1-behavsci-16-01454] Araújo D., Brymer E., Brito H., Withagen R., Davids K. (2019). The empowering variability of affordances of nature: Why do exercisers feel better after performing the same exercise in natural environments than in indoor environments?. Psychology of Sport and Exercise.

[B2-behavsci-16-01454] Avey J. B., Reichard R. J., Luthans F., Mhatre K. H. (2011). Meta-analysis of the impact of positive psychological capital on employee attitudes, behaviors, and performance. Human Resource Development Quarterly.

[B3-behavsci-16-01454] Bacevičienė M., Reviakaitė I. (2025). The relationship between nature exposure and life satisfaction in a sample of working-age Lithuanian residents: The role of nature connectedness, mindful awareness, and mental health. Visuomenės Sveikata/Public Health.

[B4-behavsci-16-01454] Banwell N., Michel S., Senn N. (2024). Greenspaces and health: Scoping review of studies in Europe. Public Health Reviews.

[B5-behavsci-16-01454] Barragan-Jason G., Loreau M., de Mazancourt C., Singer M. C., Parmesan C. (2023). Psychological and physical connections with nature improve both human well-being and nature conservation: A systematic review of meta-analyses. Biological Conservation.

[B6-behavsci-16-01454] Bertieaux D., Hesbois M., Goyette N., Duroisin N. (2024). Psychological capital and well-being: An opportunity for teachers’ well-being? Scoping review of the scientific literature in psychology and educational sciences. Acta Psychologica.

[B7-behavsci-16-01454] Brito H. S., Carraça E. V., Palmeira A. L., Ferreira J. P., Vleck V., Araújo D. (2022). Benefits to performance and well-being of nature-based exercise: A critical systematic review and meta-analysis. Environmental Science & Technology.

[B8-behavsci-16-01454] Chen F. F. (2007). Sensitivity of goodness of fit indexes to lack of measurement invariance. Structural Equation Modeling: A Multidisciplinary Journal.

[B9-behavsci-16-01454] Christiana R. W., Besenyi G. M., Gustat J., Horton T. H., Penbrooke T. L., Schultz C. L. (2021). A scoping review of the health benefits of nature-based physical activity. Journal of Healthy Eating and Active Living.

[B10-behavsci-16-01454] Cohen J. (1988). Statistical power analysis for the behavioral sciences.

[B11-behavsci-16-01454] Costello A. B., Osborne J. (2005). Best practices in exploratory factor analysis: Four recommendations for getting the most from your analysis. Practical Assessment, Research, and Evaluation.

[B12-behavsci-16-01454] Coventry P. A., Brown J. E., Pervin J., Brabyn S., Pateman R., Breedvelt J., Gilbody S., Stancliffe R., McEachan R., White P. L. (2021). Nature-based outdoor activities for mental and physical health: Systematic review and meta-analysis. SSM-Population Health.

[B13-behavsci-16-01454] Datu J. A. D., Valdez J. P. M. (2019). Psychological capital is associated with higher levels of life satisfaction and school belongingness. School Psychology International.

[B14-behavsci-16-01454] Dawkins S., Martin A., Scott J., Sanderson K. (2013). Building on the positives: A psychometric review and critical analysis of the construct of psychological capital. Journal of Occupational and Organizational Psychology.

[B15-behavsci-16-01454] Delhey J., Hess S., Boehnke K., Deutsch F., Eichhorn J., Kühnen U., Welzel C. (2023). Life satisfaction during the COVID-19 pandemic: The role of human, economic, social, and psychological capital. Journal of Happiness Studies.

[B16-behavsci-16-01454] Diener E., Emmons R. A., Larsen R. J., Griffin S. (1985). The satisfaction with life scale. Journal of Personality Assessment.

[B17-behavsci-16-01454] Dirzyte A., Perminas A., Biliuniene E. (2021). Psychometric properties of satisfaction with life scale (SWLS) and psychological capital questionnaire (PCQ-24) in the Lithuanian population. International Journal of Environmental Research and Public Health.

[B18-behavsci-16-01454] Dóci E., Spruyt B., De Moortel D., Vanroelen C., Hofmans J. (2023). In search of the social in psychological capital: Integrating psychological capital into a broader capital framework. Review of General Psychology.

[B19-behavsci-16-01454] Dudasova L., Prochazka J., Vaculik M., Lorenz T. (2021). Measuring psychological capital: Revision of the compound psychological capital scale (CPC-12). PLoS ONE.

[B20-behavsci-16-01454] Dunn T. J., Baguley T., Brunsden V. (2014). From alpha to omega: A practical solution to the pervasive problem of internal consistency estimation. British Journal of Psychology.

[B21-behavsci-16-01454] Eime R. M., Young J. A., Harvey J. T., Charity M. J., Payne W. R. (2013). A systematic review of the psychological and social benefits of participation in sport for adults: Informing development of a conceptual model of health through sport. The International Journal of Behavioral Nutrition and Physical Activity.

[B22-behavsci-16-01454] Fredrickson B. L. (2001). The role of positive emotions in positive psychology. The American Psychologist.

[B23-behavsci-16-01454] Godin G. (2011). The Godin-Shephard leisure-time physical activity questionnaire. The Health & Fitness Journal of Canada.

[B24-behavsci-16-01454] Goldberg D. P., Gater R., Sartorius N., Ustun T. B., Piccinelli M., Gureje O., Rutter C. (1997). The validity of two versions of the GHQ in the WHO study of mental illness in general health care. Psychological Medicine.

[B25-behavsci-16-01454] Hartig T., Mitchell R., de Vries S., Frumkin H. (2014). Nature and health. Annual Review of Public Health.

[B26-behavsci-16-01454] Houlden V., Weich S., de Albuquerque J. P., Jarvis S., Rees K. (2018). The relationship between greenspace and the mental wellbeing of adults: A systematic review. PLoS ONE.

[B27-behavsci-16-01454] Hsu T.-L., Barrett A. E. (2020). The association between marital status and psychological well-being: Variation across negative and positive dimensions. Journal of Family Issues.

[B28-behavsci-16-01454] Hu L., Bentler P. M. (1999). Cutoff criteria for fit indexes in covariance structure analysis: Conventional criteria versus new alternatives. Structural Equation Modeling: A Multidisciplinary Journal.

[B29-behavsci-16-01454] Huang D., Tang H., Jing L., Lei H., Li X., Koh D. (2025). Investigating the relationship between psychological capital and physical activity among university students: The mediating effects of health consciousness and health motivation. Sage Open.

[B30-behavsci-16-01454] Hussain S., Habib D. S. (2026). Urdu translation and psychometric evaluation of the revised compound psychological capital scale (CPC-12R): Evidence from intersex and transgender individuals in Pakistan. The Critical Review of Social Sciences Studies.

[B31-behavsci-16-01454] Ikeda M., Hatano K., Tanaka S., Nakahara J. (2022). Validation of the Japanese version of the revised version of the compound psychological capital scale (CPC-12R). Frontiers in Psychology.

[B32-behavsci-16-01454] Jankauskiene R., Baceviciene M. (2025). An analysis of the factors associated with the seasonal variability of physical activity in natural environments in a sample of Lithuanian adults. Behavioral Sciences.

[B33-behavsci-16-01454] Jimenez M. P., DeVille N. V., Elliott E. G., Schiff J. E., Wilt G. E., Hart J. E., James P. (2021). Associations between nature exposure and health: A review of the evidence. International Journal of Environmental Research and Public Health.

[B34-behavsci-16-01454] Kačmár P., Kušnírová K., Dudášová L., Vaculík M., Procházka J. (2022). Measuring psychological capital in the Slovak language: Validation of the revised Compound PsyCap Scale (CPC-12R_SK). Československá Psychologie.

[B35-behavsci-16-01454] Kamitsis I., Francis A. J. P. (2013). Spirituality mediates the relationship between engagement with nature and psychological wellbeing. Journal of Environmental Psychology.

[B36-behavsci-16-01454] Kaplan S. (1995). The restorative benefits of nature: Toward an integrative framework. Journal of Environmental Psychology.

[B37-behavsci-16-01454] Kessler R. C., Andrews G., Colpe L. J., Hiripi E., Mroczek D. K., Normand S.-L. T., Walters E. E., Zaslavsky A. M. (2002). Short screening scales to monitor population prevalences and trends in non-specific psychological distress. Psychological Medicine.

[B38-behavsci-16-01454] Kim M., Perrewé P. L., Kim Y. kyoum, Kim A. C. H. (2017). Psychological capital in sport organizations: Hope, efficacy, resilience, and optimism among employees in sport (HEROES). European Sport Management Quarterly.

[B39-behavsci-16-01454] Klumbiene J., Veryga A., Sakyte E., Petkeviciene J., Grabauskas V. J., Kriaucioniene V. (2015). Health behavior among Lithuanian adult population, 2014.

[B40-behavsci-16-01454] Korkmaz S., Goksuluk D., Zararsiz G. (2014). MVN: An R package for assessing multivariate normality. The R Journal.

[B41-behavsci-16-01454] Laezza L., Vacondio M., Fornasiero A., Pellegrini B., Pasini M., Brondino M., De Dominicis S. (2025). Evaluating the benefits of green exercise: A randomized controlled trial in natural and built environments assessed for their restorative properties. Psychology of Sport and Exercise.

[B42-behavsci-16-01454] Larson M., Luthans F. (2006). Potential added value of psychological capital in predicting work attitudes. Journal of Leadership & Organizational Studies.

[B43-behavsci-16-01454] Lee K., Ashton M. C., Choi J., Zachariassen K. (2015). Connectedness to nature and to humanity: Their association and personality correlates. Frontiers in Psychology.

[B44-behavsci-16-01454] Li R., Che Hassan N., Saharuddin N. (2023). Psychological capital related to academic outcomes among university students: A systematic literature review. Psychology Research and Behavior Management.

[B45-behavsci-16-01454] Liu Y., Lu L., Yao Y. (2026). Associations of psychological capital latent profiles with depression and anxiety: A cross-sectional study in Chinese college students. Public Health.

[B46-behavsci-16-01454] Lorenz T., Beer C., Pütz J., Heinitz K. (2016). Measuring psychological capital: Construction and validation of the Compound PsyCap Scale (CPC-12). PLoS ONE.

[B47-behavsci-16-01454] Lorenz T., Hagitte L., Prasath P. R. (2022). Validation of the revised Compound PsyCap Scale (CPC-12R) and its measurement invariance across the US and Germany. Frontiers in Psychology.

[B48-behavsci-16-01454] Luthans F., Avey J. B., Avolio B. J., Peterson S. J. (2010). The development and resulting performance impact of positive psychological capital. Human Resource Development Quarterly.

[B49-behavsci-16-01454] Luthans F., Avolio B. J., Avey J. B., Norman S. M. (2007). Positive psychological capital: Measurement and relationship with performance and satisfaction. Personnel Psychology.

[B50-behavsci-16-01454] Luthans F., Luthans K., Luthans B. (2004). Positive psychological capital: Beyond human and social capital. Department of Management: Faculty Publications.

[B51-behavsci-16-01454] Luthans F., Youssef-Morgan C. M. (2017). Psychological capital: An evidence-based positive approach. Annual Review of Organizational Psychology and Organizational Behavior.

[B52-behavsci-16-01454] Malinauskiene V., Leisyte P., Malinauskas R., Kirtiklyte K. (2011). Associations between self-rated health and psychosocial conditions, lifestyle factors and health resources among hospital nurses in Lithuania. Journal of Advanced Nursing.

[B53-behavsci-16-01454] Mansouri M., Lorenz T. (2025). Adaptation and validation of the Arabic version of the revised compound psychological capital scale (CPC-12R). Gruppe. Interaktion. Organisation. Zeitschrift Für Angewandte Organisationspsychologie (GIO).

[B54-behavsci-16-01454] Matukynienė K., Bacevičienė M., Jankauskienė R. (2021). Associations between nature exposure, nature connectedness, perceived restorativeness and psychological well-being in Lithuanian inhabitants. Visuomenės Sveikata/Public Health.

[B55-behavsci-16-01454] Mensah C. A., Andres L., Perera U., Roji A. (2016). Enhancing quality of life through the lens of green spaces: A systematic review approach. International Journal of Wellbeing.

[B56-behavsci-16-01454] Moortel D. D., Vos M., Spruyt B., Vanroelen C., Hofmans J., Dóci E. (2024). Psychological capital and social class: A capital approach to understanding positive psychological states and their role in explaining social inequalities. PLoS ONE.

[B57-behavsci-16-01454] Nejade R. M., Grace D., Bowman L. R. (2022). What is the impact of nature on human health? A scoping review of the literature. Journal of Global Health.

[B58-behavsci-16-01454] Newman A., Ucbasaran D., Zhu F., Hirst G. (2014). Psychological capital: A review and synthesis. Journal of Organizational Behavior.

[B59-behavsci-16-01454] Orgambídez A., Borrego Y., Cantero-Sánchez F. J., León-Pérez J. M. (2025). Relationship between psychological capital and nursing burnout: A systematic review and meta-analysis. International Nursing Review.

[B60-behavsci-16-01454] Paul F. A., Banerjee I., Ali A., Saikia P., Ganie A. U. R., Gulzar S. (2026). A systematic review of psychological capital and its relationship with mental health among undergraduate university students. Archives of Medicine and Health Sciences.

[B61-behavsci-16-01454] Peddie L., Gosselin Boucher V., Buckler E. J., Noseworthy M., Haight B. L., Pratt S., Injege B., Koehle M., Faulkner G., Puterman E. (2024). Acute effects of outdoor versus indoor exercise: A systematic review and meta-analysis. Health Psychology Review.

[B62-behavsci-16-01454] Preston A., Rew L., Young C. C. (2023). A systematic scoping review of psychological capital related to mental health in youth. The Journal of School Nursing.

[B63-behavsci-16-01454] Prieto A. J. (1992). A method for translation of instruments to other languages. Adult Education Quarterly.

[B64-behavsci-16-01454] Prochazka J., Kacmar P., Lebedova T., Dudasova L., Vaculik M. (2025). The revised Compound Psychological Capital Scale (CPC-12R): Validity and cross-cultural invariance in an organizational context. International Journal of Mental Health and Addiction.

[B65-behavsci-16-01454] Rosenberg M. (1979). Conceiving the self.

[B66-behavsci-16-01454] Sánchez J. (1982). MARDIA, K. V., J. T. KENT, J. M. BIBBY: Multivariate analysis. Academic Press, London-New York-Toronto-Sydney-San Francisco 1979. xv, 518 pp., $ 61.00. Biometrical Journal.

[B67-behavsci-16-01454] Schmitt D. P., Allik J. (2005). Simultaneous administration of the Rosenberg Self-Esteem Scale in 53 nations: Exploring the universal and culture-specific features of global self-esteem. Journal of Personality and Social Psychology.

[B68-behavsci-16-01454] Song R., Song L. (2024). The relation between psychological capital and depression: A meta-analysis. Current Psychology.

[B69-behavsci-16-01454] Spahni S., Perrig-Chiello P., Krafft A. M., Perrig-Chiello P., Walker A. M. (2018). How marital status is related to subjective well-being and dispositional hope. Hope for a good life: Results of the hope-barometer international research program.

[B70-behavsci-16-01454] Swami V., Stieger S., Voracek M., Aavik T., Abdollahpour Ranjbar H., Adebayo S. O., Afhami R., Ahmed O., Aimé A., Akel M., Al Halbusi H., Alexias G., Ali K. F., Alp-Dal N., Alsalhani A. B., Álvarez-Solas S., Amaral A. C. S., Andrianto S., Aspden T., Tran U. S. (2025). Life satisfaction around the world: Measurement invariance of the Satisfaction With Life Scale (SWLS) across 65 nations, 40 languages, gender identities, and age groups. PLoS ONE.

[B71-behavsci-16-01454] Swami V., White M. P., Voracek M., Tran U. S., Aavik T., Ranjbar H. A., Adebayo S. O., Afhami R., Ahmed O., Aimé A., Akel M., Al Halbusi H., Alexias G., Ali K. F., Alp-Dal N., Alsalhani A. B., Álvarez-Solas S., Soares Amaral A. C., Andrianto S., Stieger S. (2024). Exposure and connectedness to natural environments: An examination of the measurement invariance of the Nature Exposure Scale (NES) and Connectedness to Nature Scale (CNS) across 65 nations, 40 languages, gender identities, and age groups. Journal of Environmental Psychology.

[B72-behavsci-16-01454] Twohig-Bennett C., Jones A. (2018). The health benefits of the great outdoors: A systematic review and meta-analysis of greenspace exposure and health outcomes. Environmental Research.

[B73-behavsci-16-01454] Ulrich R. S., Simons R. F., Losito B. D., Fiorito E., Miles M. A., Zelson M. (1991). Stress recovery during exposure to natural and urban environments. Journal of Environmental Psychology.

[B74-behavsci-16-01454] White M. P., Elliott L. R., Grellier J., Economou T., Bell S., Bratman G. N., Cirach M., Gascon M., Lima M. L., Lõhmus M., Nieuwenhuijsen M., Ojala A., Roiko A., Schultz P. W., van den Bosch M., Fleming L. E. (2021). Associations between green/blue spaces and mental health across 18 countries. Scientific Reports.

[B75-behavsci-16-01454] Wicks C., Barton J., Orbell S., Andrews L. (2022). Psychological benefits of outdoor physical activity in natural versus urban environments: A systematic review and meta-analysis of experimental studies. Applied Psychology: Health and Well-Being.

[B76-behavsci-16-01454] Worthington R. L., Whittaker T. A. (2006). Scale development research: A content analysis and recommendations for best practices. The Counseling Psychologist.

[B77-behavsci-16-01454] Youssef-Morgan C. M., Luthans F. (2015). Psychological capital and well-being. Stress & Health: Journal of the International Society for the Investigation of Stress.

[B78-behavsci-16-01454] Yuan Z., Zhang X., Wang F., Jin M., Teng M., He H., Wang J. (2023). Levels of psychological capital among nurses: A systematic review and meta-analysis. International Nursing Review.

[B79-behavsci-16-01454] Zhang F., Qian H. (2024). A comprehensive review of the environmental benefits of urban green spaces. Environmental Research.

[B80-behavsci-16-01454] Zhang R., Ewalds-Kvist B. M., Li D., Jiang J. (2019). Chinese students’ satisfaction with life relative to psychological capital and mediated by purpose in life. Current Psychology.

[B81-behavsci-16-01454] Zhao H., You Q., Shi L. (2025). The relationship between physical activity and college students’ sense of security: The chain-mediated role of self-esteem and psychological capital. Scientific Reports.

